# Research Progress on Absorption, Metabolism, and Biological Activities of Anthocyanins in Berries: A Review

**DOI:** 10.3390/antiox12010003

**Published:** 2022-12-20

**Authors:** Hongkun Xue, Yumei Sang, Yuchao Gao, Yuan Zeng, Jianqing Liao, Jiaqi Tan

**Affiliations:** 1 College of Traditional Chinese Medicine, Hebei University, No. 342 Yuhua East Road, Lianchi District, Baoding 071002, China; 2 College of Physical Science and Engineering, Yichun University, No. 576 Xuefu Road, Yichun 336000, China; 3 Medical Comprehensive Experimental Center, Hebei University, No. 342 Yuhua East Road, Lianchi District, Baoding 071002, China

**Keywords:** berry, anthocyanins, absorption, metabolism, biological activities

## Abstract

Berries, as the best dietary sources for human health, are rich in anthocyanins, vitamins, fiber, polyphenols, essential amino acids, and other ingredients. Anthocyanins are one of the most important bioactive components in berries. The attractive color of berries is attributed to the fact that berries contain different kinds of anthocyanins. Increasing research activity has indicated that anthocyanins in berries show various biological activities, including protecting vision; antioxidant, anti-inflammatory and anti-tumor qualities; inhibition of lipid peroxidation; anti-cardiovascular disease properties; control of hypoglycemic conditions; and other activities. Hence, berries have high nutritional and medicinal values. The recognized absorption, metabolism, and biological activities of anthocyanins have promoted their research in different directions. Hence, it is necessary to systematically review the research progress and future prospects of anthocyanins to promote a better understanding of anthocyanins. The absorption, metabolism, and biological activities of anthocyanins from berries were reviewed in this paper. The findings of this study provide an important reference for basic research, product development and utilization of berries’ anthocyanins in food, cosmetics, and drugs.

## 1. Introduction

**Figure 1:**
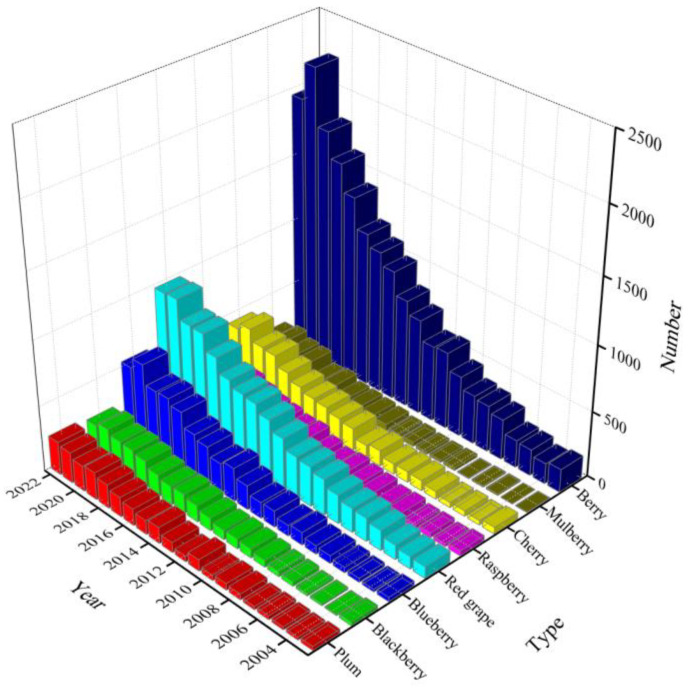
The statistics on the number of berry articles in Elsevier journals (2003–2022).

With the continuous improvement of people’s living standards, people’s awareness of healthy eating has been constantly strengthened, and many people’s eating habits have changed from traditional satiety to healthy diets. Recently, dietary guidelines in many countries have recommended increasing the daily intake of fruits and vegetables every day, among which berry fruits rich in various nutrients are widely loved by people [1]. Berries (blueberry, blackberry, plum, red grape, raspberry, cherry, mulberry, black fruit sorbus, etc.) are soft and juicy fruit, which are called the “third generation fruit” [2]. In recent years, the statistics on the number of berry-related articles in Elsevier journals are carried out from 2003 to 2022 (Figure 1). The anthocyanins content of berries from different sources are different due to different extraction methods and solvents. Table 1 summarizes the anthocyanins content of various berries [3,4,5,6,7,8,9,10,11,12].

In addition, the United Nations Food and Agriculture Organization (FAO) positioned berries as health-care fruits because berries are rich in anthocyanins, various vitamins, polysaccharides, essential amino acids, water-soluble fiber, and other active ingredients [13]. Anthocyanins, as one of the most important active components in berries, are a naturally occurring water-soluble flavonoid pigment responsible for the red, purple, and blue color of some plant tissues [14], and their structures are composed of the α- or β- linkage of anthocyanidins to a sugar moiety [15]. Growing researches have confirmed that anthocyanins in berries show various biological activities, such as beneficial antioxidant, anti-inflammatory, hypoglycemic, hypolipidemic and bacteriostasis activities, as well as inhibition of lipid peroxidation, etc. [16,17,18,19]. According to existing literature reports, anthocyanins can be used as functional active ingredients and therapeutic agents for disease prevention and treatment [20]. Therefore, anthocyanins have been paid more and more attention because of their high medical and nutritional values.

**Figure 2:**
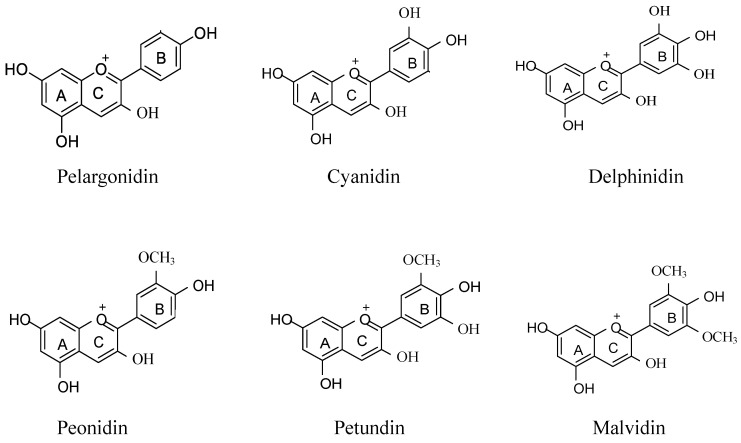
Structure of six anthocyanin monomers.

Anthocyanins are bioactive substances formed by the glycosidic bond between anthocyanidin parent and one or more glycosyl groups [21]. Chemically, anthocyanins exist as glycosides of flavylium (2 phenylbenzopyrylium) salts, but differ from them by structural variations in the number of hydroxyl groups, the degree of methylation of these hydroxyl groups, the nature and number of sugar parts attached to the molecule, and the position of the attachment, as well as the nature and number of fatty acids or aromatic acids attached to sugars [22]. The anthocyanins consist of an aromatic ring A bound to a heterocyclic ring C that includes oxygen, which is also bound by a carbon–carbon bond to a third aromatic ring B [23]. More than 600 kinds of anthocyanins have been identified in nature. There are six common anthocyanins, namely, pelargonidin (Pg), cyanidin (Cy), delphinidin (Dp), peonidin (Pn), petunidin (Pt), and malvidin (Mv) (Figure 2) [24].

Glucose (Glu), galactose (Gal), arabinose (Ara), rutinose (Rut), rhamnose (Rham), and xylose (Xyl) are the most common sugars that are bound to anthocyanins as mono-, di-, or trisaccharide forms [25]. However, the bioavailability of anthocyanins is relatively low, and the content of anthocyanins absorbed into the blood by the body only accounts for 1% of the total intake [26]. The low absorption and bioavailability of anthocyanins are closely related to their special structure, stability, and nutrient absorption characteristics in vivo [27]. Anthocyanin metabolism in humans has yet to be adequately characterised partly due to the large variation in findings in the literature [28,29,30]. This is probably the result of variations in dosages used in clinical researches [31]. Since metabolic pathways are dependent upon certain pathways that can become saturated, high doses of anthocyanins (pharmacological doses) may lead to saturation of metabolic pathways [32]. Consequently, large amounts of unconjugated parent compounds will enter the blood circulation. Additionally, the anthocyanins’ absorption efficiency cannot be fully estimated until the movement of all metabolites (gut, liver, and microorganisms) in the human body can be tracked [33,34]. Over the past century, the properties and applications of anthocyanins have been deeply investigated. Growing studies have investigated the metabolic processes of anthocyanins in the human body to explain the reasons for their low bioavailability and the mechanisms of their beneficial effects on health, aiming to identify and maximize these beneficial characteristics [35,36,37]. Nevertheless, many aspects of anthocyanin absorption and metabolism remain to be clarified. Consequently, it is necessary to clarify the absorption kinetics and metabolic modes of anthocyanins after oral administration to clarify their physiological functions as they enter the target tissues or organs in vivo. The main aim of this review is to summarize the research progress on absorption, metabolism, and biological activities of anthocyanins. The findings provide an important scientific basis for the development and utilization of anthocyanins in the future.

## 2. Absorption and Metabolism of Berry Anthocyanins

### 2.1. Absorption of Berry Anthocyanins

**Figure 3:**
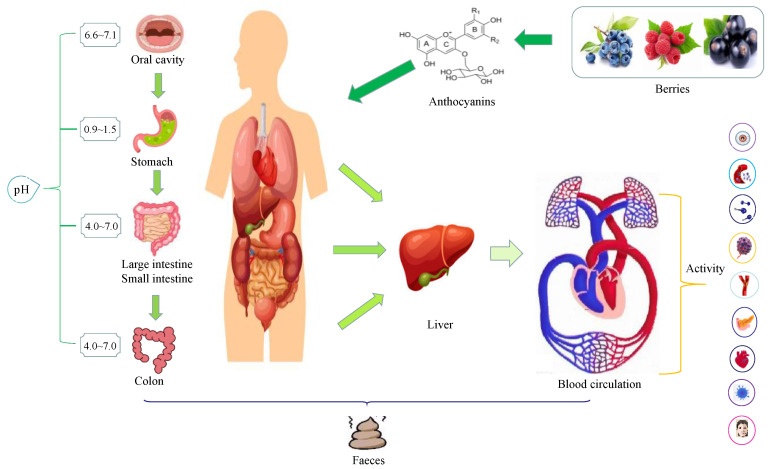
The absorption and metabolism of berry anthocyanins in the human body.

According to the Reference Dietary Nutrient Intake of Chinese Residents (2013), the specific recommended value of anthocyanins is 50 mg per day. At present, no adverse effects of anthocyanin intake on human and animal health have been found. Therefore, there is no report on the tolerable maximum intake of anthocyanins. A large number of studies have shown that the biological effects of anthocyanins vary with their intake [1,16]. The risk of type 2 diabetes was reduced when the anthocyanin intake reached 24.3 mg/day. When anthocyanin intake reached 25.3 mg/day, it could reduce the risk of myocardial infarction in women. In addition, the plasma HDL cholesterol levels significantly increased when the anthocyanin intake reached 51.5 mg/day. Furthermore, in patients with dyslipidemia and hypercholesterolemia, 320 mg anthocyanins per day for 3–6 months could improve blood lipids and vascular endothelial diastolic function. Anthocyanins are typical flavonoids, which are beneficial to human health. However, excessive intake of some flavonoids (non-anthocyanins) will also affect human health to some extent. After 24 h of administration, 60–70% of the flavonoids were eliminated by the mother, while 17% of the initial dose was still present in the fetal chamber. Previous reports suggested that the fetus might be exposed to high circulating levels of flavonoids, which might elicit toxic responses that might otherwise be innocuous to the mother [38]. Based on the average daily intake of flavonols (68 mg) and isoflavones (20–240 mg) in Asian populations, dietary exposures at these doses are not likely to cause adverse health effects [24]. To date, no human data on the long-term effects of high-dose supplementation are available. The level of flavonoids required to induce mutations and cytotoxicity may not be physiologically achievable through dietary sources. Anthocyanins are highly polar and difficult to be directly absorbed by human cells. Moreover, anthocyanins have low lipid solubility and are not easy to enter the cell through the phospholipid bilayer [26]. However, there is evidence from animal in vivo experiments that anthocyanins ingested orally are absorbed by organisms in the form of intact glycosides, which are rapidly absorbed and metabolized by various tissues and organs in the human body within 2 h after oral administration [39,40]. In general, the absorption sites of anthocyanins mainly include the oral cavity, stomach, small intestine, and colon (Figure 3).

Digestion of food, including anthocyanins, begins in the oral cavity. Upon exposure to human saliva, the degradation of anthocyanins in various berries ranged from 8 to 90%, with the mean exceeding 10% within 5 min. The pH value in the oral cavity is generally 6.6~7.1. Human oral surface epithelial cells and salivary gland terminal ducts secrete hydrolases, second stage enzymes (uridine diphosphate glucuronosyltransferase), and efflux transport enzymes required by local intestinal circulation similar to those in the small intestine, which degrades some anthocyanins into different products [41]. Walle found that the cytoplasm and oral microorganisms isolated from oral epithelial cells could degrade intact flavonoid glycosides (genistein) into aglycones, and β-glucosidase is the key enzyme for degradation in this process [42]. The oral digestion of anthocyanins is limited because anthocyanins stay in the oral cavity for a short time. The enzymes related to oral endocrine enter the alimentary canal with anthocyanins, which makes anthocyanins further digested and absorbed [43].

The undigested anthocyanins in the oral cavity enter the stomach through the upper alimentary canal. The acidic gastric juice in the stomach is a favorable environment for the stability and absorption of anthocyanins. Anthocyanins can be rapidly absorbed in the stomach without hydrolysis and appear in plasma within 30 min after ingestion [44]. The pH value of stomach environment is generally 0.9~1.5. Anthocyanins are relatively stable in the form of flavylium AH^+^ at pH ≤ 2. Approximately 1%~10% of anthocyanins are absorbed by gastric epithelial cells in a complete structure [45]. In addition, 10~20% of anthocyanins are actively transported to the blood circulation through gastric epithelial cells in the form of primary metabolites [46]. Numerous studies have indicated that the stomach is an important part of anthocyanin absorption, and the stomach’s absorption rate of anthocyanins could reach 20% [47,48]. McGhie and Walton reported that the plasma anthocyanins concentration reached the highest value within 15–30 min after rats ingested anthocyanins through gastric intubation, suggesting that the stomach plays an important role in anthocyanin absorption [49]. Fernandes et al. showed that the absorption of anthocyanins by the stomach was limited to a certain extent. High concentration of anthocyanins led to saturation of gastric absorption, which proved that anthocyanins were absorbed actively with the participation of carriers [50]. However, the mechanism of anthocyanin absorption by the stomach is not clear, and it has been reported that this absorption might depend on uridine diphosphate glucuronosyltransferase and sulfamic acid transferase in gastric tissues [51]. In addition, the absorption and transformation of anthocyanins in the stomach depends on bilirubin translocation enzymes, which take their organic anions as carriers, enter the circulatory system through the liver, and transfer to the intestine with bile after metabolism [52]. Passamonti et al. found that bilirubin translocase could effectively promote the absorption of anthocyanins in the stomach. Nevertheless, the absorption of anthocyanins in the stomach is limited because anthocyanins stay in the stomach for a short time [53].

As the longest part of the digestive tract, the small intestine plays a major role in the absorption of nutrients from food. The anthocyanins undigested by stomach tissue enter the small intestine along the lower alimentary canal. Compared with the stomach environment, the pH value in small intestine is close to neutral in the range of 4.0~7.0, while anthocyanins are prone to form quinone bases, pseudobases (B), and chalcones (C) under neutral and slightly acidic conditions, which are vulnerable to nucleophilic attack of water and are unstable [54]. Moreover, after anthocyanins enter the small intestine, anthocyanins undergo glycosylation reaction and rapid metabolism. Anthocyanins can be decomposed and converted into low molecular weight, absorbable phenolic acids, aldehydes, and other metabolites by intestinal microorganisms, which can further promote human health [55]. The activities of lactase at the edge of small intestinal villi and β-glucosidase in small intestinal microorganisms may be the basis of degradation, which can hydrolyze anthocyanins into free anthocyanins [56]. Hydrolysis of glycosidic bonds is the key to anthocyanin digestion because their aglycones are more easily absorbed than the ingested glycosides. Zou et al. studied the absorption characteristics of cyanidin-3-glucoside in the intestine through Caco-2 cell model and speculated that the absorption mechanism of anthocyanins in the small intestine might be related to transporters such as P-glycoprotein, multidrug resistance related proteins, sodium dependent glucose transporter 1, and glucose transporter 2 [57]. He et al. studied the absorption of cyanidin-3-glucoside by perfusing the small intestinal mucosa of rats. The results confirmed that cyanidin-3-glucoside was absorbed into the intestinal microvessels and into the intestinal tissue, while the individual aglycones were not measured [58]. However, cyanidin-3-glucoside glucuronic acid and sulfate are detected as intestinal metabolites. Additionally, the absorption and utilization of anthocyanins are affected by the external environment, animal age, different stress levels, and different types of glycosides bound by anthocyanins [59].

The undigested anthocyanins in the small intestine enter the colon, and the pH value in the colon is similar to that in the small intestine. The complex physiological conditions and microbial flora in the colon can destroy the ring structure of anthocyanins and degrade them into simple phenolic acids such as vanillic acid and hippuric acid [60]. Keppler and Humpf established an in vitro model based on the pig cecum. The results show that the intestinal flora could modify the structure of anthocyanins and rapidly deglycosylate and demethylate anthocyanins. The intestinal flora could degrade the anthocyanins’ ring system and remove anthocyanins’ glycosyl complexes (degrade O-glycosides and C-glycosides) [61]. After anthocyanins are metabolized by intestinal flora, the products could be absorbed by epithelial cells and enter the blood circulation to exert their effects [62]. Consequently, intestinal flora play an important role in the biotransformation of anthocyanins. Anthocyanins enter the human body to exert their biological activities through digestion and absorption in the stomach and small intestine. In addition, intestinal flora interact with anthocyanins to reabsorb them after modification or degradation. The biological activities and various health care effects of anthocyanins are jointly exerted by the parent anthocyanins and their intestinal metabolites [57]. Therefore, enhancing the stability of anthocyanins and promoting the absorption of anthocyanins in the gastrointestinal tract are of great significance to improve their bioavailability. Protocatechuic acid, ferulic acid, gallic acid, syringic acid, p-coumaric acid, and vanillic acid are the main phenolic acids produced by anthocyanins under the metabolism of microorganisms in the cecum [63]. The metabolized phenolic acid has good antioxidant, anti-inflammatory, and anti-tumor effects [64]. The absorption and metabolism of berry anthocyanins provide an important theoretical basis for their biological activities. Hence, berry anthocyanins, as a dietary source, play a role in promoting human health in two aspects [60]. (1) Anthocyanins are directly absorbed in stomach and small intestine; and (2) The anthocyanins’ decomposition products are reabsorbed in colon.

### 2.2. Metabolism of Berry Anthocyanins

Numerous studies have suggested that many flavonoids (anthocyanins, flavonols, isoflavones, dihydroisoflavones, chalcone, etc.) are widely metabolized in the human body [65]. However, less than 5–10% of the ingested parent (intact) compounds (low to medium oral doses) are excreted into the urine. It is reported that up to 52% of the oral dose of radiolabeled quercetin in human body is exhaled in the form of ^14^CO_2_. The results show that flavonoids are significantly absorbed and extensively metabolized. Although it is reported that many flavonoids have low bioavailability due to their extensive metabolism requirements, their metabolites may exist in the cycle for a long time [42]. Therefore, flavonoids have significant biological activities. Growing studies have found that anthocyanin metabolites retained their basic anthocyanin structure [66,67]. Hence, most of their biological activities may be retained. To form the working hypothesis that anthocyanins may have a metabolic fate in vivo, it is necessary to first review the basic processes related to the metabolism of flavonoids and polyphenols.

Previous studies have shown that the majority of flavonoids exist in the circulation and urine in the form of methylation, sulfation, glucuronidation, and glycosylated conjugates [68,69]. However, only 0.1–1.5% of dietary quercetin intake is not metabolized. Glucuronic acid binding is considered to be the main binding reaction in flavonoids metabolism. There are two main reasons for the widespread application of glucuronic acid pathway [25]: (1) Glucuronic acid is directly derived from glucose and stored as glycogen, therefore, it is easy to obtain; (2) Glucuronic acid has the ability of conjugation with various compounds. O-glucuronides (linkage by oxygen atom) are the most common form of glucuronide conjugation, making highly hydroxylated flavonoids the main target of glucuronidation. The glucuronylation reaction is catalyzed through UDP glucuronosyltransferase, which exists in high concentrations in the liver, intestine and kidney [70]. Among all tissues, the liver has the largest glucuronification capacity. Increasing evidences have indicated that the intestine is the initial and main site of flavonoid glucuronic acid after typical human diet intake [71,72]. Methylation seems to be the second most important conjugation reaction involving flavonoids. Methylation is driven by a group of enzymes called methyltransferases. These non-specific enzymes exist in many tissues, including liver and intestine. The most common methylation reaction related to flavonoids metabolism is O-methylation, and catechol-O-methyltransferase uses S-adenosyl methionine as a cofactor to catalyze O-methylation [73]. The liver has the highest catechol-O-methyltransferase activity and is the primary organ responsible for methylation. The hydroxylation mode of the flavonoid’s ring structure will determine the main site of methylation. Previous research has shown that quercetin was extensively methylated in humans and animals after low-dose oral administration [74]. Sulfation or glycination is also a common conjugation reaction, which plays a dominant role in the administration of low-dose phenolic drugs. Sulfation reactions are catalysed by sulfotransferases, which are a small group of cytosolic enzymes extensively distributed throughout the body [75]. They use phosphoadenosine-5′-phosphosulfate as a cofactor, and their known substrates include phenols and polyphenols (i.e., flavonoids), iodothyroxine, 4-nitrophenol, and hydroxyaromatic amine. In addition, sulfation, as a conjugation reaction, is relatively expensive in ATP and sulfate, and it is more likely to be limited by the rapid loading of ligand than glucuronization. Therefore, sulfate binding is considered to be a highly saturated pathway. It is difficult to identify flavonoid sulfide because of its relatively low concentration in blood and urine. The knowledge system on the subject of anthocyanin metabolism is far less extensive than many for other flavonoids. The metabolic results of anthocyanins remain a controversial issue. The presence of unmetabolised (parent) anthocyanin glycosides in human blood and urine has been widely documented. However, although many researchers indicate that anthocyanins are not metabolized before they are released into the systemic circulation, recent evidence suggests that this is not the case. Recently, the detection of glucuronic acid, methyl, and sulfoconjugates has been recorded, and between 68% and 80% of anthocyanins in urine are reported as metabolic derivatives [75]. The research on the identification of anthocyanins as unmetabolized parent compounds may be due to the large dose intervention on the metabolic pathway saturation, insufficient extraction procedures and detection methods, which may lead to the wrong identification [25]. The research on the identification of anthocyanins as unmetabolized parent compounds may be due to the large dose intervention on the metabolic pathway saturation, the insufficient extraction procedures and detection methods, resulting in the wrong identification [76].

## 3. Biological Activities of Berry Anthocyanins

**Figure 4:**
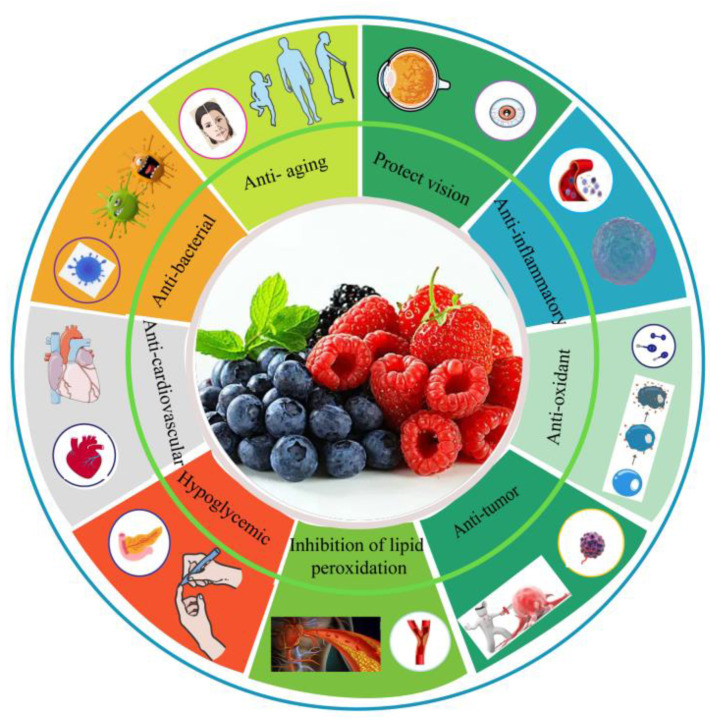
The biological activities of berry anthocyanins.

In recent years, the development and utilization of natural and harmless anthocyanins has become a hot topic in the research of phytochemistry and food science. Most berries show different colors because they contain different kinds of anthocyanins. Numerous studies have confirmed that anthocyanins, as natural food pigments, show a variety of biological activities, including protecting vision; antioxidant, anti-inflammatory and anti-tumor qualities; inhibition of lipid peroxidation; anti-cardiovascular disease properties; control of hypoglycemic conditions; and other activities [16,17,18,19] (Figure 4).

### 3.1. Protecting Vision

Today, the widespread use of smart phones, tablets and other electronic devices has led to a sharp increase in the number of patients with impaired vision in the rapidly developing Internet era. A large number of studies have shown that anthocyanins in berries could enhance visual sensitivity, which is also one of the most important biological activities of anthocyanins [77,78]. Anthocyanins are the active components with the function of protecting vision. However, the protective effect of anthocyanins on the retina and their related molecular mechanisms need to be further studied. Numerous researchers have explored the three aspects of in vitro and in vivo experiments, namely, cell, animal, and population intervention models [79]. In the cell model, retinal pigment epithelium (RPE) cells are one of the main target cells of visible light induced retinal damage. In RPE cell model, anthocyanins in berries could significantly reduce the expression of vascular endothelial growth factor and inhibit β-galactosidase activity. Huang et al. investigated the protective effect of blueberry anthocyanins extract (BAE) and its main components on the injury of human retinal capillary endothelial cells (HRCECs) induced by high glucose. The results show that BAE could reduce the levels of ROS and VEGF, and inhibit Akt pathway, ICAM-1 and NF-κB, whereas BAE could increase enzyme activities of CAT and SOD, suggesting that BAE can protect HRCECs through antioxidant and anti-inflammatory mechanisms, which may be a potential molecule for developing nutritional drugs to prevent diabetes retinopathy [80]. Yin et al. investigated the protective effect of *Vaccinium uliginosum* anthocyanins (VUA) on retinal 661W cells against microwave radiation induced retinal damage. The results suggest that VUA could decrease the apoptosis of mouse retinal photoreceptor 661W cells, stabilize the cell membrane, and reduce oxidative damage. The molecular mechanisms might be to activate Nrf2/HO-1 signaling pathway and induce HO-1 transcription and translation [81]. Increasing researchers have further explored the protective effect and molecular mechanisms of berry anthocyanins on retina in animal models (rats, rabbits, and other animals). Lee et al. studied the effect of cyanidin-3-O-glucoside (C3G) from mulberry fruit on a rat retinal degeneration (RD) model. It was found that long-term administration of C3G could structurally decrease photoreceptor damage and functionally enhance scotopic visual functions in the RD rat model induced by N-methyl-N-nitrosourea [82]. Matsunaga et al. explored whether bilberry anthocyanins and/or their main anthocyanins components (cyanidin, delphinidin, and malvidin) could protect retinal ganglion cells (RGC) from retinal damage in vivo and in vitro. The results display that bilberry anthocyanins, cyanidin, delphinidin, and malvidin could dramatically inhibit SIN-1-induced neurotoxicity and radical activation in RGC-5 and lipid peroxidation in mouse forebrain homogenates. In addition, bilberry anthocyanins and their three monomers could inhibit the NMDA-induced morphological retinal damage and increase in TUNEL-positive cells in the ganglion cell layer. Thus, bilberry anthocyanins have neuroprotective effects (partially through antioxidant mechanism) in in vivo and in vitro models of these retinal diseases [83]. Wang et al. established a model of phototoxicity in pigmented rabbits and investigated the protective effect and the possible molecular mechanisms of bilberry anthocyanins in this model. The results show that after administration of bilberry anthocyanins at dosages of 250 and 500 mg/kg/day, retinal dysfunction was substantially suppressed in terms of electroretinograms, and the reduced thicknesses of retinal outer nuclear layer. The lengths of the outer segments of photoreceptor cells were inhibited in rabbits with retinal degeneration. Bilberry anthocyanins could inhibit the proteins expression levels of Bax, Bcl-2, and Caspase-3, whereas bilberry anthocyanins could increase the antioxidant enzyme activity. Moreover, bilberry anthocyanins could inhibit the levels of IL-1β and VEGF, implying that bilberry anthocyanins can show protective effects via enhancing the antioxidant defense mechanisms, inhibiting lipid peroxidation and proinflammatory cytokines, and suppressing apoptosis of retinal cells [84]. Song et al. investigated the effects of blueberry anthocyanins (BA) on oxidative stress and inflammation in the retina of rats induced by diabetes. It was found that BA could inhibit diabetes-induced weight loss and enhance blood glucose. BA also could increase the antioxidant capacity of the retina, and decrease MDA and ROS levels. Furthermore, BA could down-regulate the levels of IL-1β and VEGF, and increase the mRNA and protein levels of Nrf2 and HO-1, suggesting that BA can protect retinal cells from diabetes-induced oxidative stress and inflammation through Nrf2/HO-1 signaling pathway [85]. With the deepening of research on retinal damage in modern medicine, increasing anthocyanins extracts and their monomers have been found to have the function of preventing retinal damage, and new methods and theories of anthocyanin extracts and monomers to protect vision have also been enriched and improved. However, there is still no systematic summary and analysis on the mechanism of different anthocyanin components on retinal damage and their relationship and differences on retinal damage. Therefore, the summary and molecular mechanism of different anthocyanin components on retinal oxidative damage still need to be further studied to provide a theoretical basis for accurate dietary nutrition supplement for protection vision.

### 3.2. Antioxidant Activity

The production and elimination of free radicals in the body is balanced under normal circumstances. However, the excessive production of free radicals or obstacles within the antioxidant system in the body lead to the imbalance of free radical metabolism [86]. Therefore, timely and appropriate supplementation with exogenous antioxidants is of great significance to prevent the occurrence of diseases and maintain the health of the body. Anthocyanins are highly reactive due to their natural electron deficiency. Anthocyanins are listed as natural products with strong antioxidant activity and show many functions, such as scavenging free radicals in the body, reducing the activity of oxidase, reducing the level of glyceride, improving the catabolism of high glyceride lipoprotein, inhibiting the absorption of cholesterol, and reducing the content of low-density lipoprotein cholesterol [87,88]. The antioxidant activity of anthocyanins is closely related to their chemical structure. The phenolic hydroxyl group on the B ring of anthocyanins is considered to play an important role in scavenging free radicals [89]. In addition, the position and type of chemical groups on the aromatic ring also affect the antioxidant activity of anthocyanins to some extent. Li et al. evaluated blueberry anthocyanin antioxidant activity by determining DPPH, ABTS^+^, and O^2−^ radicals scavenging activity, FRAP, and reducing power. The results show that blueberry anthocyanins could effectively scavenge DPPH, ABTS^+^, and O^2−^ free radicals. Moreover, blueberry anthocyanins could improve the capacity of FRAP and reducing power, indicating that blueberry can effectively exert its prebiotic activity, which is beneficial to human health [90]. Coklar and Akbulut extracted non-anthocyanin phenolic fractions and anthocyanins from *Mahonia aquifolium* berries by different solvents and further explored their antioxidant activity. The results showed that the antioxidant activity of anthocyanins was about twice that of non-anthocyanin phenolic fractions [91]. Li et al. prepared anthocyanin monomers (cyanidin-3-arabinoside, cyanidin-3-glucodise, and cyaniding-3-galactoside) from black chokeberry and evaluated their antioxidant activities. It was found that these three anthocyanin monomers could improve SOD, GSH, and CAT activity, decrease the levels of TNF-α, IL-1β, IL-6, MDA, and MCP-1, and inhibit the protein expression levels of caspase-9, suggesting that anthocyanins have potential effects on acute renal ischemia by reducing inflammation, oxidative stress, lipid peroxidation, and apoptosis [92]. Ogawa et al. evaluated anthocyanin monomer (delphinidin) antioxidant activity by lipid peroxidation and free radical scavenging capacity. The findings displayed that delphinidin showed high scavenging activity against the ·OH in a concentration-dependent manner, implying that the protective effect of *Vaccinium myrtillus* L. on HCl/ethanol-induced gastric mucosal damage may be partly attributed to the antioxidant effect of anthocyanins monomer [93]. Li et al. investigated the antioxidant activities of blueberry anthocyanins extracts via DPPH, ABTS^+^, OH free radicals and FRAP assay, and evaluated protective effect of anthocyanins extracts on acrylamide-induced toxicity in HepG2 cell model. The results showed that blueberry anthocyanins extracts could effectively scavenge DPPH, ABTS^+^, OH free radicals and improve FRAP capacity. In addition, blueberry anthocyanins extracts could improve T-SOD and CAT activities, reduce the levels of MDA, and inhibit acrylamide-induced cytotoxicity. Through the analysis of the above literature, it is found that anthocyanins could play an antioxidant role by eliminating free radicals, improving the activity of antioxidant enzymes, and reducing the damage of peroxidation to the body [94]. Therefore, anthocyanins are expected to become natural candidate drugs for the prevention and treatment of various chronic diseases. However, the molecular mechanisms of anthocyanin antioxidant activity are still unclear, and further research is still needed in the future.

### 3.3. Anti-Inflammatory Activity

Inflammation is a kind of defense response to stimulation, which is characterized by redness, swelling, heat, pain and dysfunction. Inflammatory bowel disease (IBD) is a common chronic recurrent gastrointestinal disease, affecting the health of millions of people around the world [95]. At present, researchers are trying to find a new method to replace the traditional natural therapy from terminal treatment to daily preventive health care. Anthocyanins, as a green and nutritious dietary supplement, are undoubtedly widely favored by consumers. Growing evidence has indicated that anthocyanins could effectively increase intestinal permeability and change intestinal bacterial metabolism to prevent inflammation [96].

**Figure 5:**
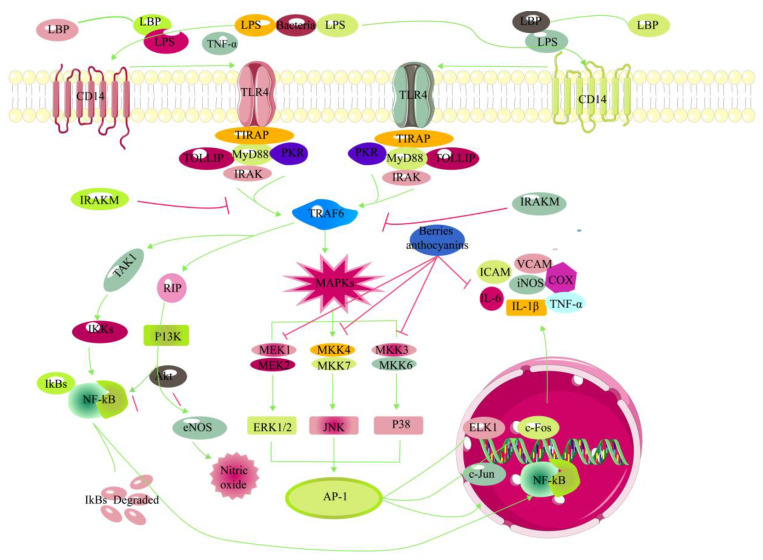
Recent studies on anti-inflammatory mechanisms of berry anthocyanins.

In recent years, with the deepening of the research on the anti-inflammatory effect of anthocyanins, researchers mainly focus on anthocyanins’ anti-inflammatory activity in vivo and in vitro. Szymanowska and Baraniak extracted and isolated anthocyanin fractions from raspberry pomace and evaluated their anti-inflammatory activity. The results displayed that four anthocyanin fractions had good anti-inflammatory activity by inhibiting the activity of lipoxygenase and cyclooxygenase 2 [97]. Chen et al. investigated the anti-inflammatory and anti-nociceptive activities of total flavonoids (TF) from black mulberry fruits. The results show that the TF contained two anthocyanins, namely, cyanidin-3-O-glucoside and cyanidin-3-O-rutinoside. TF could inhibit the levels of pro-inflammatory cytokines (IL-1β, TNF-α, IFN-γ, and NO) in the serum of mice, implying that TF have anti-inflammatory and analgesic effects, which might be related to their antioxidant activity and inhibition of proinflammatory cytokines [98]. Huang et al. investigated the protective functional role of blueberry anthocyanin extract (BAE) and its predominant constituents on high glucose-induced injury in HRCECs. The results show that BAE, malvidin, malvidin-3-glucoside, and malvidin-3-galactoside could decrease the levels of ROS and NO, increase the enzyme activities of CAT and SOD. Moreover, BAE and malvidin-3-glucoside could inhibit ICAM-1, NF-κB, VEGF, and Akt pathway, indicating that BAE can protect HRCECs by antioxidant and anti-inflammatory mechanisms. This may be a potential molecule for developing nutritional drugs to prevent diabetes retinopathy [80]. Figure 5 shows the anti-inflammatory molecular mechanism of anthocyanins.

The TLR4 is a classical inflammatory pathway. TLR4 receptors generally exist in human T cells, B cells, myocardial cells, vascular endothelial cells, adipocytes, intestinal epithelial cells, and other cells. TLR4 receptor can bind with PAMPs and activate NF-κB through signal transduction. NF-κB regulates its downstream inflammatory genes, and promotes the expression of inflammatory factors, which plays an important role in the pathogenesis of various chronic inflammatory diseases. TLR4/NF-κB signaling pathway has two pathways, namely, MyD88 dependent pathway and MyD88 independent pathway. Banach et al. studied the effects of anthocyanins on the inhibition of lipid peroxidation and the levels of inflammatory markers in RAW 264.7 cells stimulated by lipopolysaccharide (LPS). The results show that anthocyanins had good anti-inflammatory activity by inhibiting the levels of IL-1β, TNF-α, and MDA [99]. Vandevelde et al. prepared the anthocyanin-enriched fractions (AEFs) and proanthocyanidin-enriched fractions (PEFs) from strawberry and blackberry fruits, and evaluated their anti-inflammatory activity. The results display that AEFs and PEFs could inhibit the levels of ROS, IL-1β, and IL-6. In addition, AEFs could restrain the mRNA expression levels of iNOS and COX-2 [100]. Based on the above literature analysis, it is found that anthocyanins and their monomers have anti-inflammatory activity, which further emphasizes that anthocyanins can be used as a potential health product to prevent and treat various chronic diseases.

### 3.4. Anti-Tumor Activity

Malignant tumor is one of the leading causes of death in the world. At present, chemotherapy and radiotherapy are the main methods of cancer treatment. However, the clinical application of anti-tumor drugs has strong drug resistance and large side effects, which cannot achieve satisfactory efficacy [101]. Hence, there is an urgent need to develop new non-toxic side effects of multi-target natural drugs to prevent and treat tumors. Anthocyanins, as natural active substances, have undoubtedly become a hot topic in the development of natural anticancer active substances. Increasing researchers have explored the anti-tumor activity of anthocyanin extracts and anthocyanin monomers to provide a wider range of clinical drugs for the treatment of malignant tumors [102]. Existing studies have confirmed that multiple signal pathways are involved in tumor prevention and treatment. PI3K/Akt/mTOR, NF-κB/Nrf2, Bax/Bcl-2/Caspase are the three main pathways for anti-tumor activity of natural substances [103]. Thibado et al. evaluated anticancer activity of anthocyanins from bilberry and found that anthocyanins could up-regulate tumor suppressor genes, increase apoptosis in cancer cells, repair and protect genomic DNA integrity, indicating that bilberry anthocyanins can be used as natural anti-tumor drug candidates [104]. Pan et al. preformed a comparative analysis on the anti-cancer activity of anthocyanins and pyranoanthocyanidins. It was found that anthocyanidins had the strongest inhibitory effect on HeLa cells among the three anthocyanin pigments. Moreover, three anthocyanin pigments could induce cell cycle arrest at the G2/M phase and increase the protein expression levels of p53, implying that three anthocyanin pigments have good anti-cancer activity through activating p38 MAPK/p53 signaling pathway [105]. Faria et al. studied the anticancer activity of blueberry anthocyanin extract against breast cancer cells (MDA-MB-231 and MCF7 cells). The results display that blueberry anthocyanin extract could dramatically inhibit MDA-MB-231 and MCF7 cells proliferation at 250 μg/mL. Additionally, blueberry anthocyanin extract could inhibit cells invasion, enhance apoptosis, and activate caspase-3 in MCF7 cells [106]. Kim et al. investigated the anticancer activity of anthocyanins from the fruits of *Vitis coignetiae* Pulliat (AIMs) against Hep3B cells and explored the molecular mechanism of anti-tumor. It was found that AIMs could inhibit the Hep3B cell proliferation, invasion, and migration with a maximum concentration of 100 µg/mL. Moreover, AIMs could activate the activation NF-κB signaling pathway, and inhibit intra-tumoral microvessel density and the Ki67 activity of Hep3B xenograft tumors in athymic nude mice. These findings indicate that AIMs show good anticancer activity by activating NF-κB signaling pathway [107]. Xue et al. found that anthocyanins from blueberry wine residues could substantially inhibit HepG2 cell proliferation, improve intracellular ROS levels, accelerate cell apoptosis, and arrest HepG2 cells in the S phases. These results show that blueberry anthocyanins can be used to develop natural anti-tumor drugs [108]. UlIslam et al. found that anthocyanins showed potent anticancer activity by regulating the cell cycle, metabolism, survival, and proliferation, and activating PI3K/Akt/mTOR signaling pathway [109]. In summary, anthocyanin extracts and their monomers showed good anti-tumor effect. However, there are many kinds of anthocyanins and their derivatives have complex structures, which makes their anti-tumor effects different. Therefore, the anti-tumor effect and molecular mechanism of anthocyanins still need to be further studied.

### 3.5. Hypoglycemic Activity

**Figure 6:**
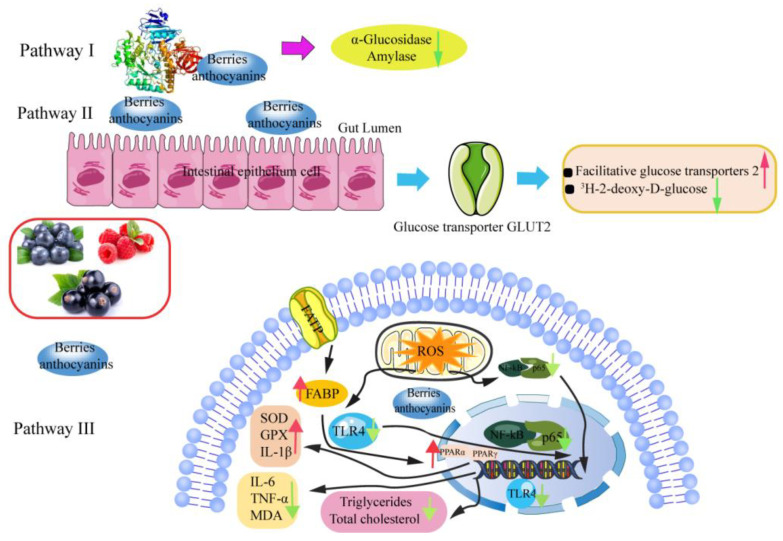
Hypoglycemic mechanisms of berry anthocyanins in enzyme activity, cell and animal models. Note: ↑ shows up-regulation; ↓ represents down-regulation.

Diabetes, as a metabolic disease characterized by hyperglycemia, has attracted extensive attention [110]. Diabetes can be divided into three major categories, namely type 1, type 2, and gestational diabetes, and type 2 diabetes accounts for more than 90% of cases. Type 2 diabetes is closely related to the impairment of β cell function and insulin resistance [111]. At present, the main treatment methods for type 2 diabetes are diet therapy, exercise therapy, oral anti-diabetes therapy, and insulin therapy. Taking or injecting insulin drugs will cause certain complications to the human body. Anthocyanins, as the natural active ingredients, have good anti-diabetes activity including reducing blood glucose, improving glucose homeostasis, and inhibiting of α-glucosidase activity [112,113]. Hui et al. explored the α-amylase and α-glucosidase inhibitory activities of blueberry extracts and investigated their inhibition kinetics. The results show that blueberry extracts could increase α-amylase and α-glucosidase inhibitory activities. Blueberry extracts showed the mixed-type inhibition on α-amylase and α-glucosidase. Moreover, the inhibitory activities of blueberry extracts on α-amylase and α-glucosidase might be driven by hydrogen bond, suggesting that blueberry can be developed into bioactive health products with anti-diabetes activities. The content of anthocyanins in berries significantly affected the regulation of postprandial blood glucose [114]. Barik et al. compared hypoglycaemic effect on high anthocyanin content black currants (BC) and low anthocyanin content green currants (GC). The results show that BC could substantially inhibit yeast α-glucosidase activity at lower concentrations than acarbose, whereas GC displayed no inhibition at the same concentrations. Moreover, BC and GC could significantly inhibit α-amylase, glucose uptake, and the mRNA expression levels of sugar transporters, implying that anthocyanins with high content in BC have the greatest impact on postprandial hyperglycemia by inhibiting digestive enzyme activities [115]. Faria et al. investigated the absorption of anthocyanins from red grape skins at the intestine using Caco-2 cells and found that anthocyanins (200 μg/mL) could inhibit 3H-2-deoxy-D-glucose uptake and increase the mRNA expression levels of facilitative glucose transporters 2, indicating that anthocyanins can be absorbed by Caco-2 cells, and interfere with their own transport and intestinal absorption of glucose to achieve hypoglycemic effect [116]. Herrera-Balandrano et al. explored the hypoglycemic and hypolipidemic effects of blueberry anthocyanin extract (BAE), malvidin (Mv), malvidin-3-glucoside (Mv-3-glc), and malvidin-3-galactoside (Mlv-3-gal) in both HepG2 cells and in a high-fat diet combining streptozotocin-induced diabetic mice. It was found that BAE, Mv, Mv-3-glc and Mlv-3-gal could markedly decrease ROS levels and improve the activity of glycogen and lipolytic decomposition enzymes. Moreover, BAE could decrease body weight of diabetic mice and improve AMPK activity, achieving the decrease of blood- and urine-glucose, triglyceride, and total cholesterol, indicating that anthocyanins contribute to the hypoglycemic- and lipid-lowering effects of blueberry extract in diabetes patients. In addition, anthocyanins may be a promising functional food or drug for the treatment of diabetes [117]. Fang et al. evaluated anthocyanins and cyanidin-3-O-glucoside hypoglycemic effect by streptozotocin and high-fat diet-induced type 2 diabetic rats. The results displayed that anthocyanins and cyanidin-3-O-glucoside could decrease the fasting blood glucose, triglycerides, total cholesterol, malondialdehyde of rats, and increase SOD activity, implying that mulberry anthocyanins have a good hypoglycemic effect [118]. Wu et al. found that blueberry extracts could significantly increase SOD activity, whereas blueberry extracts could decrease the obesity weight, TNF-α, IL-6, and NF-κB levels. The findings show that blueberry extracts, as a health product with great potential and practical value, show unique advantages in the treatment of diabetes [119]. Figure 6 shows the possible molecular mechanisms of the hypoglycemic activity of anthocyanins. All in all, there are many kinds of anthocyanins in berries, and different structures of anthocyanins have different functions. Therefore, further study is needed to explore the hypoglycemic activity of anthocyanins in vivo and in vitro.

### 3.6. Anti Cardiovascular Disease

At present, cardiovascular diseases seriously threaten human health [120]. High incidence rate, disability, and mortality are the unfortunate results of cardiovascular diseases. The levels of molecular biomarkers of cardiovascular diseases include LDL oxidation, lipid peroxidation, plasma total antioxidant capacity, and dyslipidemia [121]. Coronary heart disease, stroke, and peripheral artery disease are characteristics of several cardiovascular diseases [122]. The above diseases are caused by many factors in daily life including high cholesterol, smoke, hypertension, diabetes, age, and unhealthy diet [123]. Therefore, searching for natural drugs to prevent and treat cardiovascular diseases has become a hot topic in current research. Anthocyanins, as a dietary supplement of active ingredients, can protect cardiovascular system by inhibiting the oxidation of low-density lipoprotein (LDL) and regulating signal pathway [124]. Mohamed found that anthocyanins from mulberry could improve the total plasma antioxidant capacity, dyslipidemia and glucose metabolism, whereas anthocyanins could decrease the levels of MDA, NO, TG, LDL, and VLDL, suggesting that mulberry is becoming a dietary source of many compounds and nutrients, including anthocyanins, flavonoids, vitamins, and fibers, which can reduce the risk of cardiovascular disease [125]. Wu et al. found that three anthocyanin monomers (40 or 200 mg/kg) could markedly suppress body weight gain, decrease insulin resistance, adipocyte size, lipid accumulation, and leptin secretion. Hence, the addition of anthocyanins monomers in the diet can prevent weight gain of diet-induced obese mice [126]. Sozanski et al. evaluated the effects of anthocyanins from the fruit of *Cornus officinalis* on blood lipid level, PPARα protein expression, aortic atherosclerosis, redox status, and proinflammatory cytokines in rabbits with hypercholesterolemia. It was found that anthocyanins (100 mg/kg b.w.) could reduce in serum triglyceride levels and prevent development of atheromatous changes in the thoracic aorta. Moreover, anthocyanins could significantly increase PPARα protein expression in the liver, implying that its hypolipidemic effect may be due to the enhancement of fatty acid catabolism. Furthermore, the experimental exploration of animal models is to better verify that anthocyanins are beneficial to human anti-cardiovascular disease [127]. Qin et al. investigated the effects of berry-derived anthocyanin supplements on the serum lipid profile in dyslipidemic patients. The results show that anthocyanin supplements could increase HDL-cholesterol concentrations and decrease LDL-cholesterol concentrations. Additionally, anthocyanin supplements could decrease the mass and activity of plasma cholesteryl ester transfer protein [128]. To sum up, anthocyanins have a good protective effect on cardiovascular diseases. However, the molecular mechanism of anthocyanins’ protective effect on cardiovascular disease needs further study.

### 3.7. Other Activities

Anthocyanins, as a natural food pigment, have attracted researchers’ interest for their unique health functions. Currently, anthocyanins have received extensive attention from the scientific community and all sectors of society. In addition to the above biological activities, anthocyanins also have anti lipid peroxidation, antibacterial, and anti-aging activities. Li et al. evaluated the capacity of anthocyanins from *Aronia melanocarpa* of black chokeberry to inhibit lipid peroxidation in vitro. The results show that anthocyanins could inhibit the levels of TNF-α, IL-1β, IL-6, MCP-1, TBARS, MDA, and caspase-9 protein expression, whereas anthocyanins could increase the activities of SOD, GSH, and CAT, indicating that anthocyanins can improve acute renal failure by increasing antioxidant activity, inhibiting lipid peroxidation and promoting cell protection [92]. Denev et al. measured anthocyanin extracts from five kinds of berries (chokeberry, elderberry, black currant, blackberry, and blueberry) in terms of ORAC, HORAC, scavenging of NO, and inhibition of lipid peroxidation. It was found that anthocyanin extracts could inhibit lipid peroxidation and have the highest TRAP value (4051 μmol TE/g) and HORAC value (1293 μmol GAE/g). Moreover, anthocyanin extracts could reduce NO generation and increase antioxidant activity [129]. Silva et al. characterized the impact of anthocyanin extracts from blueberry upon the growth, adhesion and biofilm formation of several pathogens including some multiresistant bacteria. The results show that anthocyanins extracts could hinder the growth of *Staphylococcus aureus* and *Escherichia coli*, and inhibit biofilm formation and bacterial adhesion for all microorganisms tested, suggesting that anthocyanin extracts, as a natural alternative antibacterial agent, show great potential to interfere with the growth of microorganisms or hinder adhesion to the surface [130]. Liu et al. found that blueberry anthocyanins extracts (BAE) showed high DPPH, ABTS^+^, and OH radicals scavenging activity. Moreover, BAE displayed a maximum value (84.64 ± 0.35)% reduction in the biofilm biomass of *Listeria monocytogenes* and the inhibition zone given by BAE against *Escherichia coli* was 16.04 ± 0.38 mm. The findings indicate that BAE can be used as a natural antioxidant and antibacterial agent and has potential development and utilization value in the food industry [131]. Deng et al. found that the minimum inhibitory concentration of *Aronia melanocarpa* anthocyanins (AMAs) against *Escherichia coli* was 0.625 mg/mL, and the minimum bactericidal concentration was 1.25 mg/mL. Furthermore, AMAs could destroy the cell wall and membrane structure of *E. coli*, indicating that AMAs can kill *E. coli* by attacking multiple targets in bacterial cells, which is conducive to the development and utilization of AMA as a natural food preservative [132].

In addition to the above activities, anthocyanins, as a natural active ingredient, also play a certain role in human anti-aging. Abdellatif et al. explored the anti-aging activity of anthocyanins from pomegranate and evaluated the effects of anthocyanins on human skin fibroblast function and epidermal keratinocytes. It was found that anthocyanins were non irritating and might reduce skin aging. In addition, skin penetration showed a good permeability minimum of 43.16% after 210 min, indicating that pomegranate anthocyanins are a safe, stable, uniform, non-stimulating, and effective local anti-aging drug for the elderly [133]. Peixoto et al. explored anthocyanin extract from *E. precatoria* fruits about its antioxidant and antiaging properties using the model organism *Caenorhabditis elegans*. The results show that anthocyanin extract could protect the worms against oxidative stress and reduce accumulation of ROS in vivo. Additionally, anthocyanin extract could up-regulate *sod-3*:GFP and down-regulate *hsp*-16::GFP, implying that anthocyanins can regulate the development of age-related markers to achieve anti-aging effect [134]. Jo et al. investigated the molecular mechanisms underlying the effects of the anthocyanin-enriched extract (AEE) on skin appearance and condition, and found that AEE could change skin water-holding capacity, transepidermal water loss, wrinkle-related parameters, and epidermal thickness in UVB-irradiated hairless mice. AEE could decrease the mRNA expression levels of MMP, and increase TIMP and antioxidant-related genes expression levels. Furthermore, AEE could down-regulate inflammatory cytokine levels and UVB-induced phosphorylation of ERK, JNK, and p38 protein levels [135]. Wei et al. found that anthocyanins from chokeberry fruit could inhibit age-associated cognitive decline and response capacity in senescence accelerated mice. Anthocyanins improved the activities of SOD and GSH-Px, and reduced MDA production. Moreover, anthocyanins decreased the levels of COX-2, TGF-β1, and IL-1, and blocked DNA damage signaling pathway, suggesting that anthocyanins may maintain the thinking and memory of aging mice by regulating the balance of redox system and reducing the accumulation of inflammation [136]. Based on the above results, it is found that anthocyanins exist in different berries and have a variety of biological activities. They play an important role in the prevention and treatment of various chronic diseases. Therefore, anthocyanins can be regarded as a functional food, which may produce more positive results in further research.

## 4. Conclusions and Future Prospects

This paper reviews recent studies on the biological activities of anthocyanins in berries by in vivo and in vitro experiments and summarizes the evidence of the absorption and metabolism of anthocyanins as human health supplements. Growing researches have confirmed that anthocyanins show various biological activities including antioxidant, anti-inflammatory, anti-cancer, anti-cardiovascular disease, hypoglycemic, hypolipidemic, and visual protection effects, etc. The antioxidant activity of anthocyanins is the most typical biological activity. A large number of studies have shown that anthocyanins intake could improve total antioxidant capacity and might ameliorate aging caused by oxidative stress through the induction of autophagy. As in the general case of polyphenols, it is also important to consider the relationship between their absorption, metabolism, and aging. For example, when anthocyanins are administered orally, most molecules will not be transferred to the blood through antioxidant activity, which may be due to glucuronisation and/or sulfate binding in the liver and small intestine. Increasing researches have implied that a variety of anthocyanins in foods are related to aging: for example, the autoimmune theory, cross-linking theory, glycation theory, and Keap1-Nrf2 theory. In addition, anthocyanins, as heat sensitive substances, are unstable in their free forms. Anthocyanins’ metabolism is dependent on their chemical structure and is reflected in their derivatives, absorption, distribution, metabolism, and excretion. According to recent research results on competition and conjugation sequencing in transportation, the changes of microflora and the effects of other food components should also be considered, including anthocyanins dosage, intake mode, duration, digestive environment, food matrix and other factors. Cooperation among research disciplines provides unique benefits for scientific progress in this area. The use of engineering bacteria, the combination of embryonic stem cells and 3D printing technologies, and the improvements of detection techniques will greatly expand our understanding of anthocyanins metabolism, and help to maximize the health benefits of these compounds and similar compounds. The limited available data as presented suggest that anthocyanins are absorbed and transported in human serum and urine primarily as metabolites. The contribution of each individual metabolite to the reported biological activity of anthocyanins should be a focus of future research. Once the complete metabolism of anthocyanins is established their true biological activities and health effects can be explored. The extraction and purification of anthocyanins are the necessary prerequisites for the study of their biological activities. Many studies have been devoted to improving the yield and stability of anthocyanins. Increasing advanced extraction technologies including enzyme assisted extraction, ultrasound assisted extraction, ultrasound-microwave assisted extraction, aqueous two-phase assisted extraction, and ultra-high pressure assisted extraction are proposed to improve the anthocyanins yield. The above methods require strict control of ultrasonic/microwave power, extraction pressure, and enzyme concentration. Too much ultrasonic power and too long extraction time lead to the degradation of anthocyanins. Microwave selective heating will lead to local high temperature of the extract, which will cause a large amount of anthocyanins to be degraded and lose their original physiological activity. Enzymes are highly selective, specific, and expensive. Therefore, enzyme assisted extraction is not suitable for large-scale industrial extraction of anthocyanins. Existing technologies, including modification, complementary color, microencapsulation, and nanocomposite delivery have been proposed to improve the stability of anthocyanins and maintain the quality and nutritional value of products. The above techniques for improving the stability of anthocyanins have some defects, which need to be further studied. In addition, understanding and improving the bioavailability of anthocyanins are also a necessary basis for the unique and high-value utilization of anthocyanins in many fields. In the future anthocyanin research, we can focus on the following two aspects. First of all, we should explore methods to extract anthocyanins efficiently and improve the stability of anthocyanins for large-scale industrial development and utilization of anthocyanins. Secondly, we should improve the bioavailability of anthocyanins in different methods to maximize the value of anthocyanins and their related derivatives.

## Figures and Tables

**Table 1:** Anthocyanins content and extraction solvents of different berries.

Source	Type of Solvent	Total Anthocyanins Content/ Concentration	pH Range	Reference
Blueberry	Supercritical CO_2_	1.58 mg/g	1.0–14.0	[3]
Blackberry	52% ethanol	2.18 ± 0.06 mg/g	1.0–13.0	[4]
Raspberry	Deep eutectic solvent	1.378 ± 0.009 mg/g	2.0–12.0	[5]
Cherry	51% ethanol	0.41 ± 0.02 mg/g	2.0–12.0	[6]
Mulberry	70% ethanol	13.57 ± 1.30 mg/g	2.0–12.0	[7]
Strawberry	60% ethanol	38.04 mg C3GE/100 g FW	1.0–12.0	[8]
Myrica	80% ethanol	4.37 mg/g	1.0–14.0	[9]
Cranberry	52% ethanol	7.25 ± 0.02 mg/g	2.0–13.0	[10]
*Lonicera edulis*	85% ethanol	298.22 ± 1.13 mg/100 g	2.0–12.0	[11]
*Lycium chinensis*	Water-containing pectinase	19.51 ± 0.21 mg/g	2.0–13.0	[12]

## Data Availability

Data available in a publicly accessible repository that does not issue DOIs Publicly available data sets were analyzed in this study.
